# Our Newspaper as Care: Narrative Approaches in Fanon’s Psychiatry Clinic

**DOI:** 10.1007/s10912-023-09834-w

**Published:** 2024-02-26

**Authors:** Nathalie Egalité

**Affiliations:** Cleveland Clinic Lerner College of Medicine, Cleveland, OH USA

**Keywords:** Narrative, Psychiatry, Medical Humanities, Postcolonial Thought, Literary Artefacts

## Abstract

This paper argues that the newspaper *Notre Journal* enshrined the importance of narrative in the revolutionary psychiatry of its founder and editor, Frantz Fanon. Anchoring my analysis in the interdisciplinarity of the medical humanities, I demonstrate how care at Hôpital Blida-Joinville in colonial Algeria was mediated by the written word. I examine Fanon’s physician writing and editorial texts detailing the use of narrative approaches in the clinic. As an object of care, *Notre Journal*’s promotion of psychic healing, social actions, and engaged professional practice shaped the interactions and experiences of patients and staff. Printed and distributed to the wider institution, the newspaper created community—during an oppressive French Occupation and at the outset of the War of Independence—in addition to nurturing creativity, curiosity, solidarity, and accountability. Still, Fanon would come to recognize the limits of narrative methods amidst cultural oral traditions, illiteracy, and divergent attitudes about narrating the self.

## Introduction

A key figure in anticolonial theorizing and revolutionary struggle, Frantz Fanon established in 1953 the psychiatric unit newspaper *Notre Journal* (French for “Our Newspaper”). Printed and distributed every week to the wider institution of the Hôpital Blida-Joinville in Blida, Algeria, from the fifth unit (*De Clérambault Pavilion*), where Fanon was the medical director, it featured short contributions written by patients and health care providers. As editor, Fanon would briefly discuss anonymized patient experiences in the service of highlighting pressing issues, elucidate lessons applicable across diagnoses, and offer teachable moments to the entire hospital (Fanon 2018, 365). Physicians, interns, nurses, and orderlies provided complementary perspectives. Patients, who comprised most of the Newspaper Committee, fulfilled editorial, publishing, and printing duties.

I argue that *Notre Journa*l is best conceptualized as an object of care. Fanon’s stewardship of the newspaper helped shape his ideas about the role of narrative in psychiatry, patient storytelling, and writing as a social activity. As a cultural artifact, beyond its ability to represent the collective environment of its origins, *Notre Journal* remains imbued with distinctive meanings, both explicit and symbolic in nature (Berlin and Carlström 2010, 252). *Notre Journal* consecrated the therapeutic importance of reclaiming language, denied to Fanon’s patients due to mental illness but also through alienation, dispossession, and silencing inflicted by colonization.

This study focuses its literary analysis on Fanon’s editorial texts authored during his three-year tenure as editor-in-chief between December 1953 and December 1956. I examine 42 *Notre Journal* editorials authored by Fanon. These texts were recovered by Amina Azza Bekkat, translated from French to English by Steven Corcoran, and reprinted in the 2018 edition of *Alienation and Freedom,* a collection of Fanon’s psychiatric writings edited by Jean Khalfa and Robert J. C. Young (Fanon 2018). Additional primary sources consist of Fanon’s physician writing: theoretical treatment of narrative and storytelling (Fanon 2008; 2002; 1988) and reports of their application in the delivery of patient care at Blida-Joinville (Fanon 2018). Fanon’s words reveal how he conceived of *Notre Journal* as a “sociotherapeutic” intervention. The newspaper helped create a healthier institutional culture, which Fanon believed played a crucial role in patients’ recovery.

For Fanon, mental care was grounded in therapeutic healing made possible through the disalienation of human relations. “In the asylum milieu,” writes Fanon (2018, 499), “social therapy is indispensable, for it has the advantage of preserving the patients’ socialized dimension.” Fanon held that the psychiatric institution had to be transformed into a caring community, a notion impressed upon him by his colleague and mentor François Tosquelles, the Catalan psychiatry prodigy who founded the influential “school of institutional therapy.” Rather than normalizing institutions that pathologized patients and increased their psychic distress, Fanon viewed care as inextricably linked to a hospital becoming “a space where inmates (staff and ‘guests’) could work out therapies in a supportive and nurturing environment” (Gibson and Beneduce 2017, 133). Care practices went beyond the clinical sphere: interventions taking the form of agricultural, maintenance, and artisanal activities were designed to remake society, the “veritable social-therapeutic milieu” (Fanon 2018, 500). This conceptualization of caring derived from Fanon’s theoretical outlook will support the evaluation of the newspaper’s successes and shortcomings as a care practice.

I begin by reviewing the literature to situate *Notre Journal* in its historical context. Locating the newspaper in the evolution of Fanon’s intellectual thought, in the Algerian colonial psychiatry landscape, and in a tradition of written expression by psychiatry patients proves helpful in understanding its function as an object of care. My argument is supported by textual evidence on three thematic fronts: 1) narrative repair, 2) transformative care, and 3) remaking a social order on the wards. Finally, I discuss the narrative limits of *Notre Journal* in light of Fanon’s documented challenges in implementing narrative approaches.

## *Notre Journal* in Context

Fanon devoted his life to understanding and treating the suffering of patients subjected to dehumanizing treatment under colonial domination. Famous for his contributions to revolutionary struggle, Fanon’s writing would become foundational to postcolonial studies. At the time of his hire as *chef de service* to head Blida-Joinville’s psychiatric service, Fanon had recently published the influential book *Black Skins, White Masks* (Fanon 2008). Fanon had also authored scientific articles and three dramatic plays (Fanon 2018), evidence of his passion for knowledge, culture, and the arts. A paradigmatic example of Fanon’s commitment to the written word, *Notre Journal* is inscribed in a trajectory of incorporating clinical experiences in political texts to raise consciousness, most saliently in *Wretched of The Earth* (Fanon 2002)*,* deemed a blueprint for Third World independence movements.

Fanon arrived at Blida-Joinville emboldened by previous successes transforming psychiatric care environments. Early in his medical training, he observed that the social sphere was shaping his patients’ experiences of psychological distress, their responses to interventions, and the quality of their relationships with clinicians in therapeutic encounters. He developed *sociogenic theory*, a critical analysis of the individual patient situation vis-à-vis social and political institutions. A residency at Saint-Alban-sur-Limagnole’s hospital (rural south of France) brought him to study with Tosquelles. Social therapy, as it came to be known by adherents of community psychiatry, was a “humanistic approach to psychiatric medicine,” requiring “the integration of everyday life into the therapeutic process” (L. R. Gordon 2015, 79). Sociotherapeutic interventions were developed to bind the patient back into a non-pathological psychosocial frame, or what Fanon (2018, 518) described as “the effort to create a society inside the hospital itself.” Believing essential “the inter-human encounters and the practical activities in which the patient gets involved during the process of rediscovery of the ego and the world” (Fanon 2018, 294), he promoted ergotherapy (occupational therapy) as crucial to the organization of a “collective and spontaneous” life.

Rehabilitating the whole person through work activities and skills development aligned with a medical tradition of occupational therapy in psychiatric hospitals. Physician Philippe Pinel, the pioneer of humane treatments for the mentally ill in 19th-century France, recommended the curative properties of work in asylums as part of his “moral treatment movement” (Reiss 2004). The therapeutic value of individual and group occupational activities, according to Tosquelles, came from reimagining institutions to “produce new vectors of transference, different forms of identifications, and alternative social relations” (Robcis 2020, 314). Fanon’s aims for a newspaper at Blida-Joinville were sociotherapeutic. As such, individual written expression shared through the efforts of patients working together was seen as a way of transforming collectivity. Fanon previously supported the activities of Saint-Alban’s psychiatric ward journal, *Trait d’union* (Tosquelles 2015). In five of his editorials published between December 1952 and March 1953 (reprinted in *Alienation and Freedom)*, he identified existential concerns pertaining to weariness, purpose, and communicating with others (Fanon 2018, 279). He discussed common patient afflictions (insomnia, memory lapses) while advancing his reformist vision for the practice of psychiatry, such as the importance of professional training for orderlies identified as crucial first-line observers (284). Eager to showcase revolutionary psychiatric methods, Fanon deployed *Notre Journal*, in part, to document the creation of a markedly different therapeutic community. He hoped it would publicize “efforts and accomplishments” to the rest of the hospital (325). Newly invested as an administrator, Fanon wanted to depart from practices *en vogue* in French colonial hospitals. As a second-line hospital, a sizeable number of patients admitted to Blida-Joinville had been declared incurable following an internment in the Mustapha hospital in the Algerian capital (188). The delivery of services at Blida-Joinville had been organized by Antoine Porot, a major figure in Algerian colonial psychiatry, who believed that North Africans were primitive beings, “essentially vegetative and instinctive” deprived of higher cortical function (Fanon 2002, 300; Gibson and Beneduce 2017). Fanon critiqued racist science’s framing of colonized subjects as biologically inferior; he stressed that harmful stereotypes about North Africans being liars and malingerers impacted care (Fanon 1988). The hospital had a policy of separating male and female patients as well as separating them along ethnic lines between “Europeans” and “natives” (“*indigènes”*) (Fanon 2018, 188). European psychiatrists (drawn from the local colonial elite or recruited from France) did not speak or offer care in Arabic, exacerbating the cultural and class divide among the personnel.

Fanon joined the Blida-Joinville at a critical juncture in Algerian History during the early years of the War of Independence (1954–1962). A climate of mistrust reigned, with patients suspected of being insurgents or sympathizers. In *Wretched of the Earth*, Fanon would write about its disastrous effects on patient care at Blida-Joinville. Patient cases exposed how the Algerian War had given rise to severe mental disturbances, including traumatic syndromes. “The truth,” he argued, “is that colonialism in its essence was already taking on the aspect of a fertile purveyor for psychiatric hospitals” (Fanon 2002, 248). Fanon’s crisis of conscience saw him morally conflicted by a practice of medicine that implicated him in the oppression of Algerians. Fanon was so troubled by the colonial administration’s contempt for the Algerian people that he stepped down from his position in December 1956. In an open letter of resignation addressed to the Resident Minister, he condemned state-sanctioned violence (Fanon 2018). Algeria consisted of “a systematized dehumanization” where the “daily murder of humanness” was enshrined as law (434). After his expulsion from Algeria, Fanon would move to Tunisia, practice psychiatry, establish another unit newspaper (in 1958), and thereafter fully devote himself to the anticolonial cause.

In the context of Algerian censorship and repression, a newspaper featuring the writings of patients took on a potent symbolic role. And while Fanon was pleased to disseminate his groundbreaking ideas, newspapers in psychiatric hospitals were an established practice by the 1950s. Creative production by patients during asylum stints during the nineteenth century included the production of newspapers in the United Kingdom and colonial territories such as Jamaica (Fryar 2018). The literature documents the establishment of newspapers as part of recreational therapy and occupational therapy programs, providing opportunities to publish writing or develop skills in press printing (Daskalova 2021). In his study of *The Opal*, the literary journal of the New York State Lunatic Asylum between 1851 and 1860, Benjamin Reiss uncovers forerunners published beginning in 1837 (Reiss 2004, 5). Meanwhile, Louise Wannell’s study of newspapers from the United Kingdom finds that a philosophical foundation was shared between institutions, emphasizing care provided through a “routine of activity, rest, and order” in a “carefully controlled environment” (Wannell 2005, 25).

Scholars engaging in critical assessments of asylum newspapers have argued that little can be discerned about mental illness or the experience of “madness” from the patient’s perspective, or what Porter terms “medical history from below” (Porter 1985, 175). Patients were forbidden from displaying delusions or describing symptoms in their writing (Reiss 2004, 9). Instead, writing prescribed as a mode of therapy would bring patients away from fantasy to a more rational realm through a process of cultural reformation and intellectual refinement (Turner 2019). Indeed, with most of the patients writing for *The Opal* being well educated, excused from manual labor by physicians, paying privately for hospital care, and occupying the best wards, the newspaper “appealed to a residual kind of literary production and consumption wherein writing and reading were civilized, emotionally restrained activities in which the socially and intellectually elite spread their wisdom through print,” thereby primarily occupying a civilizing function (Reiss 2004, 5). Other analyses have examined instances in which patients opposed medical paternalism and institutional power through their writing, with newspapers serving as an avenue for critique (Beckman, Nelson, and Labode 2022, 182). Emily Beckman and colleagues’ study on The Dual-Diagnostic Review, published by patients at Indiana’s Central State Hospital between 1986 and 1993, provides an example of productive collaboration between patients and rehabilitative staff that aimed to foster independence and advocate for improved hospital conditions while harmonizing “with patients’ own Interests and desires” (182). The contested nature of asylums newspapers suggests the need for a critical stance on how much they fulfilled a caring function for patients.

## Narrative Repair

### Importance of language

Having patients read and write an official unit newspaper was premised on the importance of language and narration in psychiatry. Trained in neuropsychiatry, Fanon nevertheless understood that biological aspects of psychiatry provided an incomplete picture of the manifestations of mental illness. Understanding language permitted a deeper understanding of the human psyche and key social relations. Feeling constrained by “the theoretical narrowness of psychiatry” (Gibson and Beneduce 2017, 31) in addressing the problem of psychic causation, he turned to disciplines where narrative was prominent (Robcis 2020, 306), namely literature, phenomenology, history, and cultural anthropology. *Black Skin, White Masks*, originally rejected as a medical dissertation because of its unconventional transdisciplinarity (L. R. Gordon 2015, 15), examined the psychic dimensions of the basic importance of the “phenomenon of language” (Fanon 2008, 8).

Fanon’s embrace of narrative was further evidenced by his interest in psychoanalytic methods and frameworks. Adopting psychiatrist Jacques Lacan’s “intersubjectivist perspective” on the pivotal role of language in mental illness, he held that madness could not be separated from the “register of human meaning,” including the realm of the symbolic (Fanon 2018, 267). In contrast to psychiatry’s turn towards biological reductionism (Gibson and Beneduce 2017, 84), psychoanalytic methods allowed space for the patient to make sense of what was happening. Fanon’s psychiatric writings detail the role language played in identifying his patients’ disease manifestations, conceptualizing their sense of self, and shaping their attitudes toward treatment and recovery. Fond of eliciting biographical writing, Fanon’s implementation of narrative methods was “oriented toward awareness, verbalization, explanation and strengthening the ego” (Fanon 2018, 493). Therapeutic dialogue permitted a human connection from which the patient’s own power for self-determination could emerge.

### Psychic healing

The concept of *narrative repair* played an important part in shaping Fanon’s psychiatry. Conceptualizing writing as psychic healing, his editorial contributions to *Notre Journal* display his beliefs about narrative, his documented use of narrative methods with patients, and his encouragement of patients and providers to write about their experiences. Fanon ascribed a therapeutic value to patients engaging in writing. “The discovery of writing,” Fanon (2018, 315) writes in an editorial, “is certainly the most beautiful one, since it allows you to recall yourself, to present things that have happened in order and above all to communicate with others, even when they are absent.” Writing was a companion activity to narrating one’s experience of illness in a clinical setting. With *Notre Journal*, Lewis R. Gordon (2015, 82) surmises, Fanon “provided an environment that maximized the experience of agency among the patients.” On the therapeutic significance of writing, he expands: “To write means to want to be read. In the same stroke it means to want to be understood. In the act of writing there is an effort being made; muddled and vague things are combatted, surpassed. It is about not enclosing yourself in woolly dreams” (Fanon 2018, 325). Fanon put this in application in his own writing produced for *Notre Journal*, employing a “direct, topical, true” language. “Known for his richness of style honed with ancestral wounds,” Amina Azza Bekkat comments, “Fanon expresses himself here in a simple manner to facilitate understanding” (Fanon 2018, 325).

Still, Fanon conceded that storytelling skills remained difficult to acquire (Fanon 2018, 315). He labeled patients’ initial contributions to *Notre Journal* as monotonous and consisting of superficial statements (“ ‘I thank the doctor for his good care,’ ‘I would like to get out soon’ ”) (356). In later editions of *Notre Journal*, Fanon would note progress in the form of writing appropriate for the medium, with patients inspired to write about events of “collective value” (356). The Journal Committee strived to create an environment of mutual respect and recognition, in which even the feeblest attempts at writing in the newspaper were encouraged. It was the agentic act of making sense of one’s experiences that represented for Fanon evidence of narrative repair. Fanon further ascribed a therapeutic value to reading. Patients reading other contributions would develop narrative competence in addition to interpersonal skills as each person’s tone reflected their attitude and personality. Fanon describes reading as an opportunity to get to know oneself, especially as they pertain to his own nature: “The complainer,” he offers, “who always has something to criticize recognizes himself, since for him everything is bad. It is by writing and by reading what the others write that the complainer perceives that he does not think exactly like the others. Then an effort to think with measured ideas can arise in him” (326).

More importantly, Fanon’s conceptualization of writing as a social endeavor clarifies the therapeutic value of *Notre Journal*. With a view of psychic healing anchored in sociality, his editorial in the *Notre Journal*’s debut issue, titled “Memory and journal” (no. 1, December 24, 1953), is devoted to the newspaper’s role as a “*journal de bord*” (translated to a “logbook or daily diary”):

On a ship, it is commonplace to say that one is between sky and water; that one is cut off from the world; that one is alone. This journal, precisely, is to fight against the possibility of letting oneself go, against that solitude. Every day a news-sheet comes out, often poorly printed, without photos and bland. But every day, that news-sheet works to liven up the boat. In it, you are informed about the ‘on-board’ news: recreation, cinema, concerts, the next ports of call. You also learn, of course, about the news on land. The boat, though isolated, keeps contact with the outside, that is to say, with the world. Why? Because in two or three days, the passengers will meet up again with their parents and friends, and return to their homes. (Fanon 2018, 315)

He elaborates in a later issue:

The patient ought not to endure hospitalization as a kind of imprisonment, but instead as the only possible way to receive the maximum amount of treatment in a minimum amount of time. And for the entire duration of hospitalization, we must strive to keep intact the links that unite the patient with the outside world. We must insist that the patients write to their family and friends as often as possible. We must insist that the patients receive visits as often as possible. The patient’s place in society, in his or her family, must be maintained. This is why the patient has to have a social attitude: writing, receiving news and narrating are some of the most important social activities. (Fanon 2018, 321)

This was especially important given that patients were interned far from their place of residence and deprived of familiar surroundings. Breaking the isolation of patients was paramount. Building and maintaining community was an important aspect of Fanon’s approach to treating patients. Equally important was not to reduce patients’ lives to their time in the hospital or their status as psychiatric patients. A recurring theme in Fanon’s psychiatric writings was imagining a world of psychiatry outside of hospitalization for the patient.

## Transformative Care

### Material object, therapeutic habits

Furthermore, *Notre Journal*’s caring function is supported by the involvement of patients and staff in the ritual shaping of routines, experiences, and interactions at Blida-Joinville. Fanon and his colleagues’ estimation was that *Notre Journal* had a therapeutic function because the newspaper was an instrument “through which the patients could re-learn to impart meaning to the constitutive elements of an environment” (Fanon 2018, 189). As a series of weekly routines, producing *Notre Journal* involved editorial meetings (Fridays at 9:30 a.m.), sourcing information about happenings on the wards, and printing and distributing physical copies of the newspaper. Fanon conceptualized *Notre Journal* as a socio-therapeutic intervention in which patients could come together and develop new skills, all the while building and strengthening social ties. Deeming socialization as a primary objective, Fanon sought to create “friendly relations” between patients in the hospital across ethnicity, geographical origin, and religion (339). “Fanon’s project,” Bekkat clarifies, “was to make accessible to patients the creative, cultural and manual activities that might enable them to become human beings again with personal aspirations” (Fanon 2018, 312).

Among the newspaper’s goals was to describe the myriad of activities available on the ward. These included a library, sewing rooms, workshops for pottery and basket weaving, radios, film screenings, walks outside the hospital, excursions to football matches, outings to the seaside, theatre performances, artistic events, cafes, salons, dance halls, flower floats, and the celebration of religious feasts (e.g., Eid, Christmas) and public holidays (e.g., Bastille Day). In addition to keeping patients up to date, the committee solicited reports from participants so that *Notre Journal* served as a forum for reflecting on shared life at Blida-Joinville. The Newspaper Committee planned each issue, deliberating questions of format and style, such as the one-year anniversary redesign of the front page to depict a mosque typical of Algerian architecture (Fanon 2018, 339).

Fanon emphasized the therapeutic importance of routine, staying engaged with the world’s rhythms through the newspaper, and living “in time.” The rehabilitative character of participating in activities is reiterated when Fanon later writes that **“**a psychiatric institution’s first goal should be to establish, against this background of inertia and indifference, some tasks, occupations and timetables” (Fanon 2018, 336). In Fanon’s characteristic open manner, his discussion of the changes underway at the newspaper provides information on logistics and habits surrounding the production of *Notre Journal*. The editorial published in the second year (no. 36, August 25, 1955) touches upon the Printing Committee, the physical location of the printing room, the patients involved in the printing process, and the volume of *Notre Journal*’s final print run. The second year of *Notre Journal*’s run would bring an increase in printing capacity with the acquisition of a “genuine printing facility” (336). “Now,” Fanon implores, “that the journal hopes to come out with a total of four pages, the instruction is formal: everyone must write. The hospital, as the sociologists say, has gone from the oral phase to the written phase” (337).

### Engaged professional practice

Fanon’s editorials articulated his vision for how caregivers ought to treat patients. An analysis of *Notre Journal* reveals its use as a teaching tool, combining guidance drawn from clinical observations with reprinted excerpts of lectures and training manuals (e.g., Paul Bernard’s psychiatric handbook) (Fanon 2018, 313). For Fanon, restoring trust in the encounter between the patient and doctor in the colonial context was imperative. In contrast to European attitudes about mental health, Fanon noted that the cultural attitudes of Maghrebi Muslims involve extending social trust to those afflicted with illness: “the collectivity never adopts a distrustful or aggressive attitude toward the patient himself” (423). Linguistic barriers created the need for an interpreter, which made patients suspicious, physicians impatient, and communication between the two difficult (367). Believing a climate of trust to be necessary for psychic healing, Fanon created spaces in which the therapeutic relationship could be rehabilitated.

*Notre Journal* functioned as an object of care through the promotion of a more engaged professional practice. Transforming caring relationships was aided by writing on changing how caregivers perceived their own roles. Fanon (2018, 338) says: “Meeting the obligation to understand, day-in day-out, the people with whom we come face-to-face and who, more precisely, are entrusted to us, is one of the most important elements of the profession of nursing.” Attuned to tensions in the professional hierarchy, Fanon stressed the importance of cordial relationships between providers to improving patient care. Discipline was a recurrent theme in Fanon’s treatment of professional practice. Admonishing the propensity for disciplinary action, Fanon educated orderlies on the phenomenon of transference, which can occur when they are tasked with enforcing norms. “We are dealing,” he writes in his rejection of universals, “with very determinate persons and as therapists we have to take these persons, in all their nuances, into consideration; we must necessarily adapt to each boarder” (347). Fanon promoted patient-centered care based on a recognition of their unique needs as whole persons. Ahead of his resignation from Blida-Joinville, he would devote his remaining three editorials to orderlies in a symbolic show of confidence in their ability to take over the reins (314). Fanon’s final *Notre Journal* editorial (no. 52, third year, December 20, 1956) reiterates the consequences of “disastrous attitudes” on the well-being of patients.

## Remaking a Social Order on the Wards

### Creating a community

*Notre Journal* fostered a sense of community at the Blida-Joinville hospital in addition to encouraging creativity, solidarity, and accountability. M. Cohen, a patient and member of the Journal Committee, testifies in the pages of *Notre Journal* to its utmost value. “It provokes,” he writes, “the boarders’ personalities and gives them back their souls. It is a powerful link between us all; it is also a vehicle of understanding and a remarkably effective agent of connection” (Fanon 2018, 328). Above all, the creation of community on the wards was geared towards patients’ recovery. For Fanon, the wards had to be therapeutic in their very structure: daily activities were organized to create an atmosphere conducive to sociability. Of the emergence of a social order on the wards, Fanon writes: “It is within spontaneous or institutionalized groups that the boarders, through the play of preferences and wishes, respond and commit. As members of the group, the staff never adopt the aspect of observers or censors. Quite to the contrary, a staff member’s nuanced and balanced engagement enables the group to become organized and a source of life” (340). Fanon further impressed the importance of being welcoming to visitors; he urges the receptionist, as the first point of contact with the unit, to be agreeable, friendly, and courteous (324). This was important given the establishment of links with other groups of psychiatric patients. Blida-Joinville was “part of the twenty psychiatric hospitals involved in developing constellations of socially structured activities for the hospitalized patient” (324). The provision of progressive enrichment hinged on fostering cohesion, team spirit, and healthy group dynamics. Editorial texts outlining strategies for community building, along with articles detailing opportunities for engagement, served to reiterate the narrative possibilities of *Notre Journal*.

Under Fanon’s stewardship, *Notre Journal* can be distinguished from asylum newspapers by its emphasis on the therapeutic importance of cultural considerations at Blida-Joinville. Fanon’s earlier psychiatric writings yielded analyses incorporating cultural dimensions, while his critical treatment of patient cases theorized about the role of culture in mental illness. His revolutionary thought addressed the specific contours of cultural alienation for his Algerian patients, the necessity of the cultural context in psychiatry’s explanatory apparatus, and the cultural moorings of psychiatry itself. A scientific article published by Fanon and Jacques Azoulay on the men’s ward noted its heterogeneous character, consisting of ethnic French, Arabs, Kabyles, Chaouis, Moroccans, and Mozabites (Fanon 2018, 365). Consequently, *Notre Journal* reflected attempts at developing Blida-Joinville as a therapeutic community receptive to the cultural diversity of patients. Tolerance and respect for each culture became a recurring theme in reporting on the social organization of the ward. Fanon describes in *Notre Journal* the upcoming visit by the Mufti of Blida to meet with patients and preside over a service at the mosque as “the first encounter between official Muslim religion and our patients” (324). Fanon called for greater integration with *Notre Journal* with other activities, believing that “Our Journal ought to reflect the social life within the establishment” (345). Special efforts to provide culturally appropriate care were meant to mark a stark contrast to life outside the hospital. This therapeutic structure was a countermeasure to colonial Algeria’s social organization, which Fanon theorized as causative of mental illness.

### Doing right by each other

Correspondingly, a recurring theme in *Notre Journal* was living well and doing right by each other. Fanon proclaimed his steadfast focus on the “essential condition of correct social relations” (Fanon 2018, 328). The newspaper published testimonies of recognition, like the patients’ gratitude towards a driver who ferried “forty boarders at a pleasant speed enabling them to appreciate and savour the excursion” (324). Cultivating character remained a preoccupation for Fanon in his implementation of sociotherapy. Of the banality of everyday actions , Fanon argued: “The hero is not one who performs a dazzling feat and goes to bed considering he’s done enough. The hero is rather one who gets through his or her task with conscientiousness and love. Every day, without haste but also unremittingly, he weaves his work with precise order” (336). Fanon’s view of good character was strongly tied to work. Likewise, there was a powerful sense of ethical behavior in care providers promoted in *Notre Journal*. “Each time we disregard our profession,” Fanon writes, “each time that we give up our attitude of understanding and adopt an attitude of punishment, we are mistaken” (346). The newspaper thus contributed to shaping patients and staff as model ward citizens.

In the remaking of a social order on the wards, it became important to refine the rationale and applicability of rules and norms. Fanon (2018, 341) explains: “When two people meet and decide to live together, they come to agreement on a certain number of points. Between them some things are permitted, and some things are prohibited. The advised things pertain to morality and the prohibited things to the law. Every social group implies and requires, among other things, moral authorities, and legal authorities.” Publishing the draft list of rules for the Sports Committee, he outlines a vision of sport as curative of “abnormal behavior,” which meant eliminating “offensive words, insults, provocative gestures, assaults” (341). While it was primordial to prepare patients for reintegrating into society, Fanon nevertheless felt conflicted about the extent to which rules and norms served a therapeutic purpose. Registering and penalizing behavior outside commonly established norms was “to make sure its authors are thwarted and shamed, this is to force them to account for their actions, to give them a sense of responsibility, to facilitate their aptitude to defend their point of view, to present it in front of the group of their fellow patients” (341). Fanon came to believe that spelling out rules created a climate of punishment that was inconsistent with psychic recovery. Fanon concluded his tenure at Blida-Joinville with this realization: “We may now see that formulating disciplinary rules and regulations at a psychiatric hospital is a therapeutic absurdity and that this idea must be abandoned once and for all” (348).

### A culture of transparency

Another value that characterized Blida Joinville was being able to speak openly about problems arising on the ward. *Notre Journal* helped foster a culture of transparency*,* beginning with patients writing in the newspaper to bring attention to problematic aspects of their daily experiences. Fanon encouraged patients to express themselves about the food available to them: patients’ perceptions of the food they eat ought to occupy a “role in the concrete and daily preoccupations of this hospital” (Fanon 2018, 332). Fanon’s position communicates that the reflections and criticisms of patients are appropriate and welcome by the staff. Fanon was forthright on administrative issues, such as costs, inefficiencies, overcrowding, and the public hospital system. In a spirit of openness, Fanon invited patients to speak on the newspaper: “Why is it that, for ten years now, so many journals have appeared in psychiatric hospitals? What is the real importance of these journals? What use is a journal in a hospital and, more precisely, in a psychiatric hospital?” (338). However, no answers to these questions were found in future editorials analyzed in this study. Furthermore, there were no published instances of patients commenting on the conduct of clinicians or criticizing the delivery of care. This aligns with scholarship questioning the ability to truly represent patients’ voices and the impossibility of taking on medical power.

Keen to impress on patients the need to have the “right attitude,” Fanon often employed an optimistic tone. “We are pleased,” he writes, “to observe the ever-widening social relations at our establishment. And we hope that these efforts will continue and enable us always to forge further ahead” (Fanon 2018, 336). Conceding the vulnerability of the institution to be weakened—“the constant danger of vitiation”—Fanon engaged in critical reflection on the psychiatric hospital and one’s place in it:

You have to place yourself at the heart of the institution and interrogate it. If it is a generous source, it must enable multiple personalities to be manifest in it. It has to make possible interminable and fruitful encounters. It has to be multiplied constantly. It has to be at the disposal of its members, at their service. If it does not radiate, if it fails to achieve its essential duty, which is constant dialogue between its members, if it permits ‘collective monologue,’ and if, lastly, it does not foster its members’ responsibility, then its time is up. One is on the wrong path. (Fanon 2018, 334–5)

Editors Jean Khalfa and Robert J.C. Young invite a re-reading of Fanon’s editorials that emphasizes his preoccupation with the fight against colonialism and political revolution in political organizations (Fanon 2018, 334, fn. 15).

## Narrative Limits

Despite Fanon’s lofty ambitions, *Notre Journal*’s impact in remaking a social order conducive to recovery from psychiatric illness was muted. The Journal Committee came to realize that few patients were aware of the existence of *Notre Journal* during its first months of existence, an indifference which received editorial treatment (no. 42, October 7, 1954). Due to linguistic and cultural barriers, *Notre Journal* suffered from a low readership and limited engagement from the wider patient population. Thus, the newspaper exercise had a minimal impact on patients. Fanon likewise confronted the limits of the written word as a therapeutic modality. Difficulties initially encountered in the use of narrative approaches at Blida-Joinville nevertheless provided Fanon and colleagues with valuable learning opportunities.

A newspaper in the French language was ill-suited to reach Algerian patients. Fanon and Charles Géromini summed up literacy levels on the Muslim ward as follows: “Out of the two hundred and twenty patients in our unit, only five knew how to read and write in Arabic and two, how to read and write in French. The others were illiterate” (Fanon 2018, 370). This was evident when, by the sixth month, only one Muslim patient contributed an article to *Notre Journal* (360). Fanon failed to recognize patients’ low literacy levels, which excluded patients from the newspaper’s activities. Culture was seldom written; teaching was carried out orally through speech (356). Yet, he sought to understand the crucial role played by *talebs* (“public writers of letters,” “tasked with reading and writing for the rest of the group”) and the itinerant storyteller (“who goes from village to village spreading news and stories from folklore,” “relating the events of previous centuries, and thereby ensures a cultural liaison between the different regions”) (371). As detailed in a scientific article about the implementation of sociotherapy on the Muslim ward, Fanon and Géromini came to understand cultural differences that posed sizeable obstacles to using Western approaches in Algeria. Introducing a newspaper without considering the specificity of the local context constituted an imposition of colonial ways of knowing. Interestingly, Fanon had written at length about the alienation brought on by the French language for colonized people, the power that accompanied the mastery of language, the resulting inferiority complexes, and the dangers of Eurocentrism in the application of psychiatric methods (Fanon 2008). At the same time, while administering patient care with greater cultural sensitivity, he failed to see how his Western approach betrayed fundamental differences with Algerian patients’ understandings of their world and the community at large. The expectation that patients articulate an illness narrative might have tacitly encouraged a construal of the self as an individual psyche detached from the communal and social realms. Fanon likewise did not recognize his own biases vis-à-vis the expression of internal mental states for public consumption using the written word.

Fanon faced additional limits in using narrative methods maladapted to the provision of psychiatric care in Algeria. Insights into North African culture helped explain why the patients were reluctant to sing, play a role, or perform on a stage during the festivities on the ward outside of traditional familial or religious contexts (Fanon 2018, 368). Initially, so, too, did the narrative work introduced by Fanon appear abstract to patients. Attempts to administer psychological tests developed with Western patients found dramatically different results with female Muslim patients. Fanon and Géromini wrote of the Thematic Apperception Test (TAT): “Most often we obtain only a dry enumeration. No guiding line is extracted. No structure emerges. Narrative is non-existent. There is no stage, no drama” (429). Rejecting both cultural relativism and racist explanations that North Africans were fundamentally lacking in imagination, they found the TAT to be culturally determined. Of the failure to capture patients’ personality changes, they write: “The dynamisms flowing within Maghrebin society, the lived experience of the surrounding European world, the Muslim’s marginalized existence, which leads to a scotomization, a disinterest, the cultural truth, ought to have been thematized. Our patient’s inadaptation is the correlate of the method’s inadequacy” (430). Mindful of situating narrative repair in a specific cultural context, Fanon would go on to develop methods of doing narrative repair work (e.g., pioneering the non-Eurocentric TAT) that were culturally appropriate for his patients.

## Conclusion

These study results inform appraisals of the limits of narrative methods in medicine and the broader medical humanities (Woods 2011; O’Mahony 2013). Evaluating the explicit and symbolic meanings of narrative approaches calls for an analysis that contextualizes narration in histories, cultures, politics, and societies. Asylum newspapers, particularly from colonial settings, testify to an indissociable link between caring and engrained ideas about superior ways of knowing. In his editorial marking *Notre Journal*’s first anniversary of publication, Fanon (2018, 330) bemoaned “the distance that exists between those who write and those who read.” He welcomed the opportunity to open *Notre Journal* to the hospital community without considering his biases or obstacles to including all patients. Although heralded as a conduit for connection, Western narrative constructs imposed on Algerians created and sharpened divisions. Orthodox ideas about the written word undermined the respect of culture and communal life usually upheld as sacrosanct in the provision of sociotherapy. Accordingly, this analysis of Fanon’s past efforts in running a newspaper lays bare pitfalls of care interventions through writing that remain favored today. Similar studies of practices presumed to confer therapeutic benefits are needed to scrutinize the use of narrative methods in medicine.

Reading *Notre Journal* through a lens of care highlights how clinicians must reconcile hopes with disappointments. Fanon’s reflections are valuable in that they give weight to therapeutic failures. In detailing his missteps and false starts, Fanon honors a commitment to the scientific attitude necessary for corrective innovations. “The real itself,” writes Avery F. Gordon (1997, 11), “and its ethnographic or sociological representations are also fictions, albeit powerful ones that we do not experience as fictional, but as true.” Fanon knew that voices resonating together in *Notre Journal* generated fictions about life on the psychiatric wards in colonial Algeria. Theorizing the effects of social determinants on the health of his patients and advocating for an expanded notion of care, Fanon reimagined and reformed the hospital as a space for healing. Despite its muted reception, *Notre Journal* outlines truths: the concrete changes instituted by Fanon in service of a different psychiatry.
